# Two Hemocyte Lineages Exist in Silkworm Larval Hematopoietic Organ

**DOI:** 10.1371/journal.pone.0011816

**Published:** 2010-07-28

**Authors:** Yuichi Nakahara, Yasushi Kanamori, Makoto Kiuchi, Manabu Kamimura

**Affiliations:** Division of Insect Science, National Institute of Agrobiological Sciences, Tsukuba, Japan; Katholieke Universiteit Leuven, Belgium

## Abstract

**Background:**

Insects have multiple hemocyte morphotypes with different functions as do vertebrates, however, their hematopoietic lineages are largely unexplored with the exception of *Drosophila melanogaster*.

**Methodology/Principal Findings:**

To study the hematopoietic lineage of the silkworm, *Bombyx mori*, we investigated *in vivo* and *in vitro* differentiation of hemocyte precursors in the hematopoietic organ (HPO) into the four mature hemocyte subsets, namely, plasmatocytes, granulocytes, oenocytoids, and spherulocytes. Five days after implantation of enzymatically-dispersed HPO cells from a GFP-expressing transgenic line into the hemocoel of normal larvae, differentiation into plasmatocytes, granulocytes and oenocytoids, but not spherulocytes, was observed. When the HPO cells were cultured *in vitro*, plasmatocytes appeared rapidly, and oenocytoids possessing prophenol oxidase activity appeared several days later. HPO cells were also able to differentiate into a small number of granulocytes, but not into spherulocytes. When functionally mature plasmatocytes were cultured *in vitro*, oenocytoids were observed 10 days later. These results suggest that the hemocyte precursors in HPO first differentiate into plasmatocytes, which further change into oenocytoids.

**Conclusions/Significance:**

From these results, we propose that *B. mori* hemocytes can be divided into two major lineages, a granulocyte lineage and a plasmatocyte-oenocytoid lineage. The origins of the spherulocytes could not be determined in this study. We construct a model for the hematopoietic lineages at the larval stage of *B. mori*.

## Introduction

Insects combat invading pathogens and parasites by a combination of cellular and humoral defense reactions [1]. Blood cells play key roles in cellular defense reactions such as phagocytosis, encapsulation and nodule formation [2], [3], [4]. They are also involved in humoral defense by producing the precursor of a melanization enzyme phenoloxidase (pro-PO) [5], [6], [7] and antimicrobial peptides [8], [9].

Lepidopteran blood cells are generally classified into five major subsets based on morphology and functions [3], [4], [10], [11] (Supplementary data Fig. S1). Prohemocytes are accepted as a multipotent precursor cell giving rise to other subsets [12]. Granulocytes are involved in recognition of non-self, phagocytosis and encapsulation [4], [11]. Plasmatocytes adhere to and spread over foreign bodies and wounds [13], [14], and are the main capsule-forming hemocytes [2], [4]. Oenocytoids produce pro-PO [5], [6], [15], and release it into the hemolymph by rupturing after wounding [16], [17] (Supplementary data Fig. S1). The functions of the spherulocytes are unknown [4], [11], and some species including *Pieris rapae* and certain strains of *B. mori* lack this hemocyte subset [18], [19].

Insect hematopoiesis has been well-studied in *Drosophila melanogaster* (Diptera), and many similarities between vertebrates and insect have been demonstrated regarding the molecular mechanisms regulating hemocyte differentiation [20], [21], [22]. For Lepidoptera, hemocyte differentiation has been analyzed, albeit mainly by histological observation and physiological experiments, in *Bombyx mori* (Bombycidae), *Euxoa declarata* (Noctuidae), *Pseudoplusia includens* (Noctuidae) and *Manduca sexta* (Sphingidae). Thus far, the following has been reported: i) during embryogenesis, granulocytes arise from head mesoderm, and plasmatocytes are derived from thoracic mesoderm which sites correspond to the two pairs of hematopoietic organs [23], ii) the larval hematopoietic organ (HPO) is mainly a source of prohemocytes and plasmatocytes [14], [24], [25], [26], [27], although all subsets are present to some extent [28], [29]; iii) prohemocytes give rise to other hemocyte subsets [12]; iv) circulating hemocytes undergo mitosis, with the exception of oenocytoids [19], [25], [30], [31].

In the present study, we examined *in vivo* and *in vitro* differentiation of hemocyte precursors from *B. mori* HPO, in order to clarify relationships among the five hemocyte subsets. *B. mori* HPO gave rise to granulocytes, plasmatocytes, and oenocytoids, but not spherulocytes. Furthermore, we found that functional plasmatocytes involved in cellular defense have the potential to differentiate into oenocytoids involved in humoral defense. Therefore, *B. mori* hemocytes consist of two lineages with the capability of differentiating toward either granulocytes or oenocytoids. Taking all the results in the present study and past findings together, we propose a model for hematopoietic lineages at the larval stage of *B. mori*.

## Results

### *Ex vivo* differentiation of HPO cells into plasmatocytes, oenocytoids, and granulocytes, but not into spherulocytes

**Figure 1:**
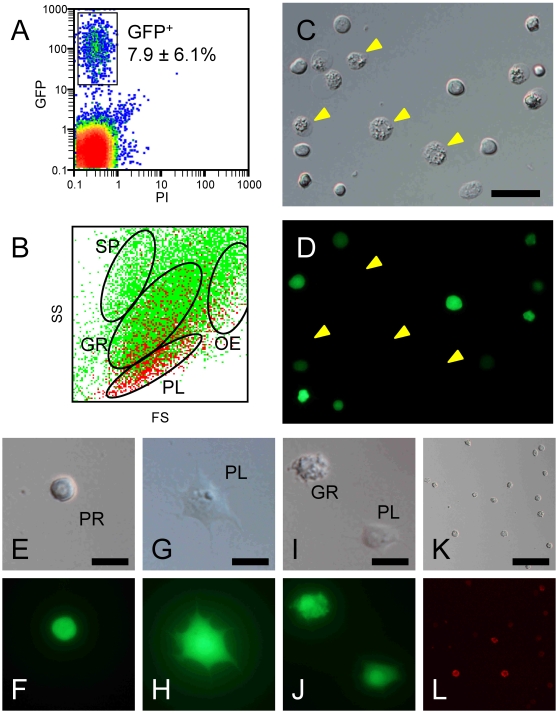
*Ex vivo* differentiation from progenitor cells in HPO. CecB-GFP HPOs were enzymatically dispersed and injected into the hemocoel of non-transgenic larvae. Five days later, collected hemocytes were analyzed (A, B) and GFP-expressing cells were sorted (C–L). A: two-dimensional plots with PI/GFP of whole collected hemocytes. B: two-dimensional plots with FS/SS of whole collected hemocytes. Green and red dots are of GFP negative and positive cells, respectively. Clusters of spherulocytes (SP), granulocytes (GR), plasmatocytes (PL), and oenocytoids (OE) are gated according to Nakahara et al. (2009). C, D: All sorted cells fluoresce bright green, except for oenocytoids (yellow arrowheads) that had collapsed immediately after sorting. Among the GFP-expressing cells, prohemocytes (E, F), plasmatocytes (G, H), and granulocytes (I, J) were also observed. The sorted GFP+ cells were stained with anti-granulocyte antibody (K, L). Bar  = 20 µm (C), 10 µm (E, G, I), 40 µm (K).

To determine whether HPO cells can give rise to all of the morphotypes observed in the circulation, we conducted implantation experiments. The transgenic line CecB-GFP [32], in which GFP is expressed under the control of the *B. mori* cecropin B gene promoter, is a useful tool for these experiments, because all hemocyte subsets fluoresce green (Supplementary data Fig. S2), although no spherulocytes were found in half of the individuals due to individual variation (see Materials and Methods). HPOs from the CecB-GFP larvae were enzymatically dispersed and injected into L5D0 larva of the standard line, and the resulting hemocytes were recovered 5 days later (Supplementary data Fig. S3). A part of GFP-expressing cells (GFP+) was observed in aggregated cell masses. In circulating hemocytes, percentages of free GFP+ cells account for 7.9±6.1% (n = 3) (Fig. 1A). Their events were distributed dominantly in the gate for plasmatocyte (57.7±4.5%), then in the gate for granulocyte (27.9±4.8%) and oenocytoid (13.5±2.8%) (Fig. 1B). A negligible level of the GFP+ events was observed in the gate for spherulocyte (0.8±0.2%), which might be noises or contaminations of granulocyte.

GFP-expressing cells (GFP+) collected by flow cytometry were comprised predominantly of plasmatocytes and oenocytoids in morphological criteria, although the latter had already ruptured and collapsed, turning dark just after sorting (Fig. 1C,D) because they are very unstable *in vitro* (see Supplementary data Fig. S2). Examined closely under the microscope, the GFP+ population was found to contain prohemocytes, plasmatocytes, oenocytoids, and granulocytes, but not spherulocytes (Fig. 1E-J). Although we repeated this experiment several times using totally more than 30 C145/N140 recipients and more than 600 CecB-GFP donors, half of which had an ability to produce spherulocytes (see Materials and Methods), no spherulocytes expressing GFP were ever identified. This indicates that spherulocytes can not be differentiated from implanted HPO cells. The identity of the granulocytes was confirmed by immunohistochemistry using an anti-granulocyte monoclonal antibody. Approximately 20% of the GFP+ cells were stained with this antibody (Fig. 1K,L), despite the fact that typical granulocytes containing small granules were not seen in large numbers.

### *In vitro* differentiation of HPO cells into plasmatocytes and oenocytoids

**Figure 2:**
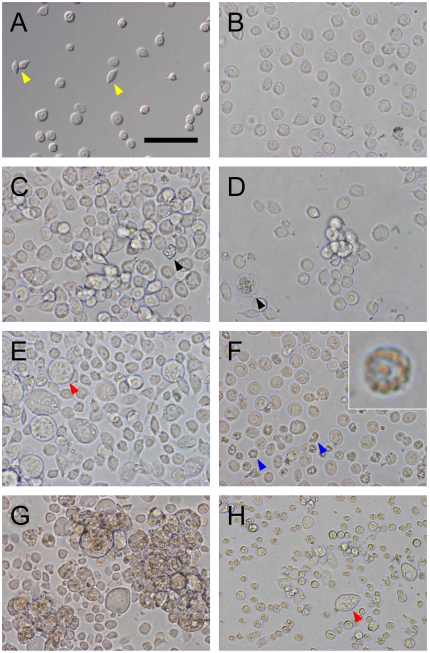
*In vitro* culture of *B. mori* hemocyte-progenitor cells. A: dispersed HPO cells viewed under a differential interference microscope. B: these cells were seeded into 24-well multiplates at a density of 2×105 cells/500 µl medium/well. C–H: the cells were cultured in medium containing 3% larval plasma (C, E, G) or plasma-free medium (D, F, H). Cells were viewed at 24 h (C, D), 72 h (E, F), and 168 h (G, H). Yellow arrowheads: spindle-shaped plasmatocytes. Black arrowheads: a collapsed oenocytoid and a spherulocyte, which had probably contaminated the HPO. Red arrowheads: oenocytoids appearing during culture. Blue arrowheads: shrunken cells (inset of panel F is a magnified image of a shrunken cell). All figures are at the same magnification (bar  = 50 µm).

To investigate the hemocyte subsets derived from larval HPO, enzymatically-dispersed HPO cells were cultured and morphological changes were recorded. At the start of culture (Fig. 2A,B), most cells were uniform in size (7–10 µm diameter) and were round-shaped, very similar in appearance to circulating prohemocytes (Fig. S1D). Spindle-shaped plasmatocytes were also observed. When the enzymatically-dispersed HPO cells were cultured with 3% larval plasma, most cells had a plasmatocyte-like appearance within a day (Fig. 2C). Cell numbers increased 4.2 times at day 2 and 5.1 times at day 4 (n = 3). In parallel, cell size increased to 11.6±2.7 µm in diameter at day 2 and to 17.7±7.9 µm at day 4. Particularly, large circular cells 20–40 µm in diameter with amorphous inclusions appeared on days 3–4 (Fig. 2E). These cells were very similar to circulating oenocytoids (Fig. S1H) both in size and appearance. The percentage of the oenocytoid-like cells accounted for 4.0±0.9% of the total cells on day 4 (n = 3). We observed masses of small prohemocyte-like cells, surrounded by spread plasmatocyte-like cells and a small numbers of the large oenocytoid-like cells (Supplementary data Fig. S4). These images suggest that the prohemocyte-like cells actively proliferated during culture and differentiated into the plasmatocyte-like and oenocytoid-like cells. After that, the cultured cell began to aggregate (Fig. 2G).

In contrast, in plasma-free medium, the enzymatically-dispersed HPO cells did not proliferate vigorously during culture (Fig. 2D). Three days later, many cells appeared damaged and shrunken (Fig. 2F). These cells contained several large granules in their cytoplasm but were different from typical granulocytes or spherulocytes, although a small number of living granulocyte-like cells were also found (see next section). At day 7, oenocytoid-like cells appeared, four days later than seen in cultures with larval plasma (Fig. 2H).

Differentiation into spherulocytes was never observed in any of the culture experiments with or without larval plasma.

### *In vitro* differentiation of HPO cells into granulocytes

**Figure 3:**
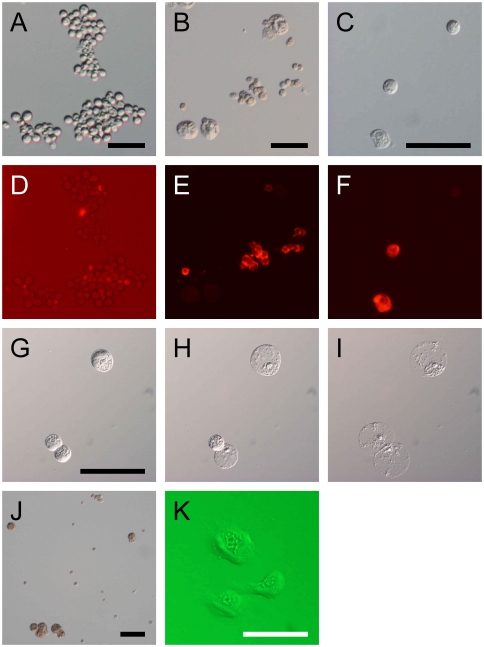
*In vitro* differentiation into granulocytes and oenocytoids. A–F: Immunostaining with anti-granulocyte antibody mAb#13 of dispersed HPO cells (A, D), circulating hemocytes from L5D1 larvae (B, E) and HPO cells cultured in plasma-free medium for 7 days (C, F). The photograph shown in (D) was intentionally overexposed in order to detect very weak signals. G–I: Collapse of cultured cells similar to oenocytoids. HPO cells cultured in plasma-containing medium for 7 days were transferred to a glass slide and viewed at 0 min (G), 5 min (H), and 60 min (I). Two of three cells collapsed within 5 min, and the third was unchanged until 50 min later, but finally collapsed at 60 min. J: the cultured cells were incubated in 1% DOPA saline for 2 h. Large cells (arrow heads) turned brown in color. K: cells having properties of both plasmatocytes and oenocytoids were found in the culture of HPO cells.

Although granulocytes were rarely observed in cultures of dispersed HPO with larval plasma, a small number of granulocyte-like cells appeared when HPO cells were cultured without plasma. These were stained with granulocyte-specific antibody [9] to determine whether HPO cells really gave rise to granulocytes. We found that both undissociated HPO and dispersed HPO cells showed very weak staining (Fig. 3AD), compared to circulating hemocytes (Fig. 3BE). This suggests that HPO contain few, if any, mature granulocytes, but the weak signal might indicate the presence of immature granulocytes. In contrast, after 4 days cultured in plasma-free medium, certain cells did stain strongly with the antibody (Fig. 3CF). These results indicate that premature granulocytes or uncommitted hemocyte precursors in HPO had indeed differentiated into mature granulocytes during culture. In plasma-containing medium, on the other hand, there were very few cells stained by the antibody, perhaps because a large number of plasmatocytes diluted out the small numbers of granulocytes.

### *In vitro* differentiation into oenocytoids

The large cells observed at later culture time points had the appearance of oenocytoids (Fig. 2E,H). To confirm this, we looked for collapsing behavior and melanization of the cells, which are characteristics of mature oenocytoids in the hemolymph.

At culture day 7, cells were collected by gentle pipetting and transferred onto a glass slide together with medium. Some of the large cells quickly collapsed (Fig. 3G,H) similar to the way in which circulating oenocytoids burst to release prophenoloxidase (pro-PO) immediately after wounding [14], [16], [17]. However, one of the 3 cells shown in Fig. 3G-I showed delayed collapse, taking approximately 1 h, suggesting differing reactivity to inflammatory factors inducing collapsing behavior.

When cultured cells were fixed and incubated with L-DOPA, the large cells changed color to brown due to melanization (Fig. 3J), indicating that they contained large amounts of pro-PO. This strong pro-PO activity is a hallmark of oenocytoids. Taking their appearance and pro-PO activity together, we conclude that they are indeed mature oenocytoids.

### Differentiation from plasmatocyte to oenocytoid cell

During the culture of HPO cells, we found cells having features of both plasmatocytes and oenocytoids, i.e. asymmetric spreading behavior (adhesive character) and copious intracellular contents (Fig. 3K), suggesting that plasmatocytes and oenocytoids belong to the same lineage. If so, the question arises as to, whether oenocytoids differentiate directly from hemocyte precursors or via a form of plasmatocyte. To test the latter hypothesis, we performed *in vitro* culture experiments with pure populations of functionally mature plasmatocytes.

**Figure 4:**
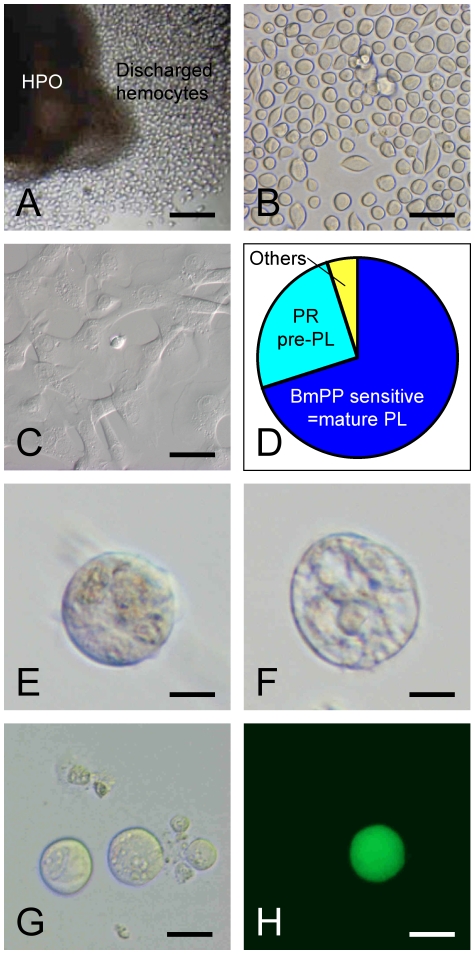
*In vitro* differentiation from plasmatocyte to oenocytoid. When *B. mori* HPO was cultured in plasma-containing medium, a large number of hemocytes were discharged (A). Discharged hemocytes were spherical or spindle-shaped (B), and approximately 70% of them underwent marked morphological changes after PP treatment [14] (C, D). Non-adherent cells, including immature plasmatocytes and other subsets (D), were washed away. The spread cells, i.e. functional plasmatocytes, were collected and cultured in medium containing larval plasma. Seven days later, oenocytoid-like cells can be seen (E, F). Likewise, functional plasmatocytes from CecB-GFP line were collected, and cultured with discharged cells from non-transgenic larvae. After 7 days, GFP expressing oenocytoid-like cells can be seen (G, H). Bar  = 0.1 mm (A), 40 µm (B, C), 10 µm (E, F), 20 µm (G, H).

To collect the plasmatocytes, HPOs with wing imaginal were made to release hemocytes by culturing in medium containing 10% larval plasma [24] (Fig. 4A,B). Approximately 70% of the cells released from HPO under these conditions were functional plasmatocytes that reacted to the insect cytokine paralytic peptide (PP), resulting in tight adhesion to the culture vessel (Fig. 4C,D). The remaining cells, insensitive to PP, were washed away. The adherent spread plasmatocytes reverted to a spherical shape after treatment with 50 mM EDTA, which caused them to detach. After washing, the plasmatocytes were further purified by repeating the PP treatment. We confirmed microscopically that all cells adhered to and spread out on culture vessels. These purified functional plasmatocytes were then cultured in MGM-450 medium containing 3% larval plasma. Almost all cells still rapidly spread out, but some reverted to round shape several days later. After 10 days, we observed round cells with copious intracellular contents (Fig. 4E) and others that had already collapsed, like oenocytoids (Fig. F). Thus, we conclude that mature plasmatocytes had differentiated into mature oenocytoids *in vitro*.

When culture was started from 10,000 plasmatocytes, we observed less than ten typical oenocytoids (N = 3). The differentiation ratio was thus <0.1%. This value is much smaller than that obtained using the enzymatically-dispersed HPO cells (4%) and we consider that this value is underestimated compared with the actual differentiation ratio *in vivo*, because almost all plasmatocytes tested in the *in vitro* experiments were forced to spread by PP addition or larval plasma repeatedly. These strong stresses might have turned off the differentiation switch of the plasmatocytes.

This experiment was repeated using the CecB-GFP line. In addition, we examined the ability of supporting cells to promote differentiation. A pure population of functional plasmatocytes from the CecB-GFP line was cultured on a sheet of spread-plasmatocytes from the standard line. At day 10, GFP-expressing cells were found to be large in size, like oenocytoids (Fig. 4G,H). In contrast to our expectation, however, no enhancing effect of supporting cells was observed.

## Discussion

Our goal in this study was to elucidate the hematopoietic lineages in the silkworm. Insect hemocytes are of dual origin [23], [33], [34]. The first population develops at embryonic stages and multiplies in the circulation at larval stages. The second population is produced in the mesodermally-derived HPO of larva. In Lepidoptera, HPO is mainly regarded as a source of prohemocytes and plasmatocytes [14], [24], [25], [26].

Thus far, the differentiation ability of plasmatocyte has been controversial. Hinks and Arnold [35] suggested that plasmatocytes give rise to oenocytoids, whereas Yamashita and Iwabuchi [12] suggested that plasmatocytes might be the terminally-differentiated form. In the present study, we showed that plasmatocytes could differentiate into oenocytoids in *B. mori* (Fig. 4). One of the common characteristics shared between them is pro-PO activity. Immunohistochemistry using anti-prophenoloxidase antibody demonstrated that only plasmatocytes and oenocytoids contain this enzyme [5]. Pro-PO1 and pro-PO2 genes of *B. mori* are expressed in newly-discharged hemocytes (mainly plasmatocytes) and circulating oenocytoids but not in granulocytes, whereas pro-PO2 gene was expressed also in spherulocytes [9]. Similarities between plasmatocyte and oenocytoid in reactivity with antibodies raised against whole hemocytes are also demonstrated in *P. includens* [36]. In addition, an inflammatory cytokine, paralytic peptide (PP), evoked morphological changes of both hemocyte subsets, namely, plasmatocyte spread on culture dishes [13], [14], [37] and oenocytoid collapse [16, Nakahara et al., unpublished data] immediately after its addition. Perhaps these changes may be caused by identical cellular reactions, i.e., polymerization of actin, but the flexibility of these cells may be different, resulting in quite different responses. Thus, we speculate that oenocytoids are aging plasmatocytes specializing in producing certain proteins including pro-PO, although this hypothesis should be accessed *in vivo*. As far as we know, there is no other cell that alters its function so drastically.

We also showed that HPO cells including prohemocytes could give rise to granulocytes (Fig. 2). This finding does not mean that all granulocytes are derived from larval HPO. We assume that a large part of granulocytes in circulation is embryo-derived like as *E. declarata* and *M. sexta* [23], [31], because the number of granulocytes released from HPOs is much smaller than circulating ones [24]. Single cell culture of circulating prohemocytes resulted in the production of plasmatocytes and granulocytes at a similar rate [12]. However, commitment to granulocyte generation in HPO seems suppressed, because these cells were rarely observed in the organs or discharged cell populations. Self-renewal of hemocyte stem cells and their commitment to differentiation depends largely on their microenvironment, referred to as the stem cell niche [38]. In *B. mori* HPO, a stem cell niche corresponding to the *Drosophila* posterior signaling center [39], [40] could also be present, although it has never been identified. Prohemocytes that moved away from the niche might then be able to differentiate into granulocytes.

Differentiation from HPO cells to spherulocytes was not observed either *in vitro* or *in vivo*. Yamashita and Iwabuchi [12] demonstrated that prohemocytes could change into spherulocytes via a granulocyte-like form rather than directly. In the present study, only a small number of granulocytes were derived from HPO *in vitro*. Therefore, it might result in little opportunity for their differentiation into spherulocytes. Alternatively, the spherulocytes may have a different origin, for example, the embryonic hemocyte population. The finding that spherulocytes show unique gene expression profiles among the four differentiated hemocyte subsets in hemolymph and HPO cells [9] supports this hypothesis.

**Figure 5:**
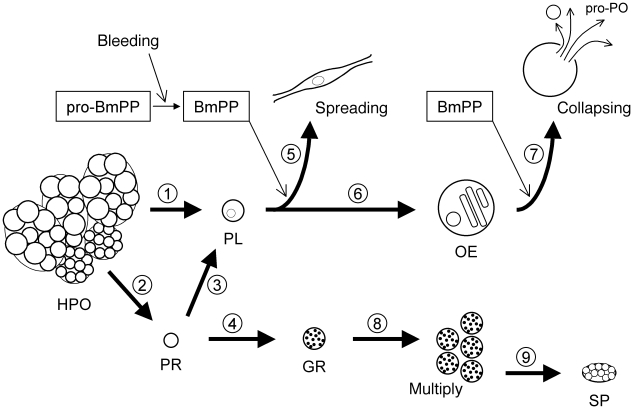
Schematic of a hypothesis for hemocyte differentiation in *B. mori* larva. 1,2: *B. mori* HPO discharges hemocytes, of which (1) approximately 70% are functional plasmatocytes with (2) the remaining cells putative prohemocytes [14]. 3,4: Prohemocytes discharged into hemolymph could give rise to (3) plasmatocytes and (4) granulocytes [12]. 5: When *B. mori* larvae are injured, pro-PP is processed into the active form [46] and induces spreading behavior of plasmatocytes [14]. 6: Without inflammation caused by the PP, plasmatocytes can differentiate into oenocytoids (Fig. 4). 7: Oenocytoids are also activated by PP, and release their cellular contents [17] (Supplementary data Fig. S1) including enzymes involved in melanization. 8: Granulocytes differentiated from prohemocytes multiply by mitosis [41], [42]. 9: Some of the granulocytes may differentiate into spherulocytes [12]. HPO: hematopoietic organ. PR: prohemocyte. PL: plasmatocyte. OE: oenocytoid. GR: granulocyte. SP: spherulocyte.

Taking all the results in the present study and past findings together, here we propose a hematopoietic lineage model for the larval stage of *B. mori* (Fig. 5). *B. mori* HPO consist of two types of islets, compact and loose islets, which contain immature cells (prohemocytes) and morphologically-mature hemocytes, respectively [28], [29]. In the HPO, the commitment seems largely biased toward plasmatocytes. Plasmatocytes in HPO are not yet functionally mature, and gain spreading-ability only after discharge into the hemolymph [14]. Approximately 70% of discharged cells are functional plasmatocytes sensitive to PP [14]. PP-insensitive cells have rounded morphology and lack conspicuous intercellular contents, which is a characteristic of both prohemocytes and unspread plasmatocytes. They cannot be distinguished by morphology, because threshold size differences between them are unclear [24]. Therefore, we propose that the PP-insensitive cells consist of a mixture of uncommitted prohemocytes and precursor cells committed to plasmatocytes that have not yet acquired the ability to respond to PP. Discharged prohemocytes are at least bipotential precursor cells that give rise to plasmatocytes and granulocytes [12]. When *B. mori* larvae are injured, plasmatocytes are recruited to cover the wound and encapsulate invaders. Without inflammation, plasmatocytes may increase in size, lose adhesive ability (flexibility of cell structure), and become oenocytoids producing a set of proteins involved in immunity. Whether the transformation is a spontaneous process or regulated by external signals remains to be determined. Oenocytoids burst and release their contained proteins after receiving PP stimuli. Granulocytes are in another lineage different from the plasmatocyte-oenocytoid lineage. Granulocytes multiply by mitosis [41], [42], and some of them might differentiate into spherulocytes [12], although this was not confirmed in the present study.

This model for *B. mori* (Bombycidae) consists with other studies for lepidopteran hematopoiesis. In *E. declarata* (Noctuidae), plasmatocytes and oenocytoids derived from HPO, while granulocytes and spherulocytes proliferate by multiplication of circulating hemocytes [31], [35]. Granulocyte and plasmatocyte represent distinct lineages also in *M. sexta* (Sphingidae) [23], [26]. Gardiner and Strand [36] suggested close-relationship between plasmatocytes and oenocytoids, while granulocytes were related to spherulocytes in *P. includens* (Noctuidae). These models indicate that lepidopteran hematopoietic lineages are highly conserved across different families, and whether it could be expanded to different insects belonging to other orders are of future interest. In *D. melanogaster*, bi-potential precursor cells giving rise to lamellocytes and crystal cells, counterparts of plasmatocyte and oenocytoid in Lepidoptera, were suggested to be existing in lymph gland [43]. This may imply a similarity in hematopoietic lineages between Lepidoptera and Diptera.

## Materials and Methods

### Silkworms

A hybrid of C145 and N140 (C145/N140), referred to as the standard line, was used unless otherwise indicated. The transgenic *B. mori* line, CecB-GFP [32], was a kind gift of Dr. K. Taniai. For both lines, larvae were reared on a commercial artificial diet (Silkmate®, Nihon Nosan Kogyo, Yokohama, Japan), under a 12-h light-dark photoregime at 25°C. For simplicity, the larval stage is expressed as, for example, L5D1 for day 1 of the 5th (final) larval stadium.

Hemocytes were classified into the 5 morphotypes, prohemocytes, plasmatocytes, granulocytes, spherulocytes and oenocytoids (Supplementary data Fig. S1 and S2) by morphological criteria, according to [28], [30].

Whether each individual has spherulocytes is determined genetically by at least two major gene loci [19]. All individuals of the C145/N140 strain have spherulocytes and their population accounted for around 10% of the total hemocytes [24]. In contrast, a preliminary observation showed that about half of the CecB-GFP individuals (55.8%, n = 52) used in this study lacked spherulocytes. The population of spherulocytes in the CecB-GFP individuals that have this hemocyte subset accounted for around 10% of the total hemocytes as in C145/N140 strain.

### *Ex vivo* differentiation

The HPOs were collected from 40 fore imaginal wing discs of L5D1 CecB-GFP larvae. After washing with serum-free Grace's medium (Gibco™, Invitrogen, Carlsbad, CA), the organs were dispersed using 0.5 U/ml of dispase® (Godo Shusei, Tokyo, Japan) in Grace's medium for 30 min, and passed through a cell-strainer (Falcon® 8235, BD Biosciences, Franklin Lakes, NJ). After washing twice with Pringle's saline, 1×10^5^ cells per 20 µl of the saline were injected into an L5D0 non-transgenic (C145/J140) larva through the second abdominal proleg. The needle wound was ligated with cotton thread. The recipients were reared on an artificial diet under a 12-h light-dark photoregime at 25°C. Five days later, approximately 100 µl of larval hemolymph was collected into 1 ml of chilled anticoagulant buffer (98 mM NaOH, 186 mM NaCl, 17 mM EDTA, and 41 mM citric acid, pH 4.5) containing 1 ng/ml of propidium iodide (PI), and passed through a cell-strainer (Falcon® 8235). The samples were analyzed by flow cytometry and GFP-positive cells were sorted.

### *In vitro* culture of HPO cells

The HPOs from L5D1 larvae were dispersed using 0.5 U/ml of dispase® in Grace's medium for 30 min, and passed through a cell-strainer (Falcon® 8235). HPO cells were then washed twice with MGM-450 medium [44], seeded at 1×10^4^ to 2×10^5^ cells/500 µl medium/well in MGM-450 medium with or without 3% larval plasma into 24-well multiplates (Falcon® 3047, BD Biosciences) and cultured at 25°C for 7 days. *B. mori* larval plasma was prepared as previously described [14].

### Plasmatocyte collection

L5D1 HPO culture in MGM-450 medium containing 10% larval plasma for 48 h, resulted in the discharge of approximately 50,000 hemocytes per organ [14], [24]. Of these, about 70% were induced to adhere to the culture vessel by paralytic peptide (PP), an insect cytokine inducing spreading behavior of plasmatocytes [14], [45]. Nonadherent cells were discarded. Adherent plasmatocytes were then treated with 50 mM EDTA in Pringle's saline (54 mM NaCl, 27 mM KCl, 14 mM CaCl2, 22.2 mM dextrose) for 15 min to detach them. After washing to remove any trace of EDTA, plasmatocytes were treated with PP again, and we confirmed that all cells adhered to and spread out on the culture vessel. Plasmatocytes were again detached using 50 mM EDTA, washed with serum-free MGM-450 medium, and cultured at 1×10^4^ cells/500 µl medium/well in MGM-450 medium containing 3% larval plasma at 25°C.

### Melanization of oenocytoids

Hemocytes were washed twice with chilled Pringle's saline and fixed in 0.25% glutaraldehyde in Pringle's saline for 20 min. After washing twice, they were reacted in 0.1% DOPA (Sigma, St. Louis, MO) for 1 h at ambient temperature [15].

### Immunostaining

Cells were washed with Pringle's saline, fixed in 4% paraformaldehyde in PBS overnight at 4°C, and blocked with 1% bovine serum albumin in PBS. Anti-granulocyte monoclonal antibody [9] and secondary antibody (goat anti-mouse IgG antibody, rhodamin-conjugated: Roche Diagnostics, Basel, Switzerland) were then added sequentially. After washing twice for 15 min, cells were viewed under fluorescent microscopy and also examined by flow cytometry after passing them through a cell-strainer (Falcon® 8235).

### Flow cytometric analysis and cell sorting

Cells were analyzed and sorted on an EPICS ELITE (Beckman Coulter, Fullerton, CA) equipped with an argon laser (488 nm), and data were compensated, analyzed, and presented by EXPO32™ software (Beckman Coulter), according to Nakahara et al. [9]. The machine was standardized with fluorescent beads and detection sensitivities were adjusted to baseline using intact hemocytes from a non-transgenic line. A sample of the sorted cells was reanalyzed to confirm their purity. Gate for each hemocyte subset was determined according to the previous study [9].

## Supporting Information

Figure S1 Hematopoietic organ (A–C) and circulating hemocytes (D–K) in B. mori (standard line). A: Hematopoietic organ (HPO) attached to imaginal fore wing disc from L5D1 larva. B: an illustration of HPO (corresponding to panel A). C: an HPO separated from imaginal wing disc. Bars are 0.2 mm. Circulating hemocytes were classified into 5 morphotypes: prohemocytes (D), granulocytes (E), spherulocytes (G), oenocytoids (H), and plasmatocytes (J), by morphological criteria according to Akai and Sato [28], [30]. One hour after separation, granulocytes and plasmatocytes transformed into the spread form (F and K, respectively). Soon after isolation, oenocytoids collapsed to release pro-PO (I).
(5.88 MB TIF)

Figure S2 Hemocytes from CecB-GFP larvae. A,B: flow cytometric analysis. A: granulocytes and plasmatocytes are dominant in silkworm, and were roughly divided on two-dimensional plots with FS/SS [9]. B: 97% of granulocytes (GR) and 83% of plasmatocytes (PL) express GFP. C–F: CecB-GFP hemocytes viewed under a differential interference microscope (upper) and a fluorescent microscope (lower) immediately after isolation (C–H) and a few minutes later (I, J). PL and GR fluoresce bright green (D, F). An oenocytoid (OE) initially looks bright (G, H) but soon after isolation collapsed and turned dark (J: the same frame as panel H, 3 min later). Spherulocytes (SP) fluoresce green in the nucleus and cytoplasm but not in the spherules. All photographs are at the same magnification (bar  = 10 µm).
(2.49 MB TIF)

Figure S3 Hemocytes derived from HPO cells after in vivo differentiation. HPOs from CecB-GFP larvae were enzymatically dispersed (A) and injected into non-transgenic larvae. Five days later, cells were recovered and viewed under a differential interference microscope (left) and a fluorescent microscope (right). Cells derived from implanted HPO cells expressed GFP (B). C: GFP-expressing plasmatocyte. D: GFP-expressing oenocytoid. E: GFP-expressing granulocyte. Bar  = 40 µm (A, B), 10 µm (C, D, E).
(3.47 MB TIF)

Figure S4 Culture of HPO cells. Enzymatically-dispersed HPO cells were cultured with 3% larval plasma for 4 days. Black arrowheads: a mass of small prohemocyte-like cells. Blue arrowheads: spread plasmatocyte-like cells. Red arrowheads: large oenocytoid-like cells. Bar  = 50 µm.
(8.38 MB TIF)
