# Supplementary figures and images for: Two Hemocyte Lineages Exist in Silkworm Larval Hematopoietic Organ
Source: PLoS One. 2010 Jul 28;5(7):e11816. doi: 10.1371/journal.pone.0011816 (PMC2911379; doi:10.1371/journal.pone.0011816)

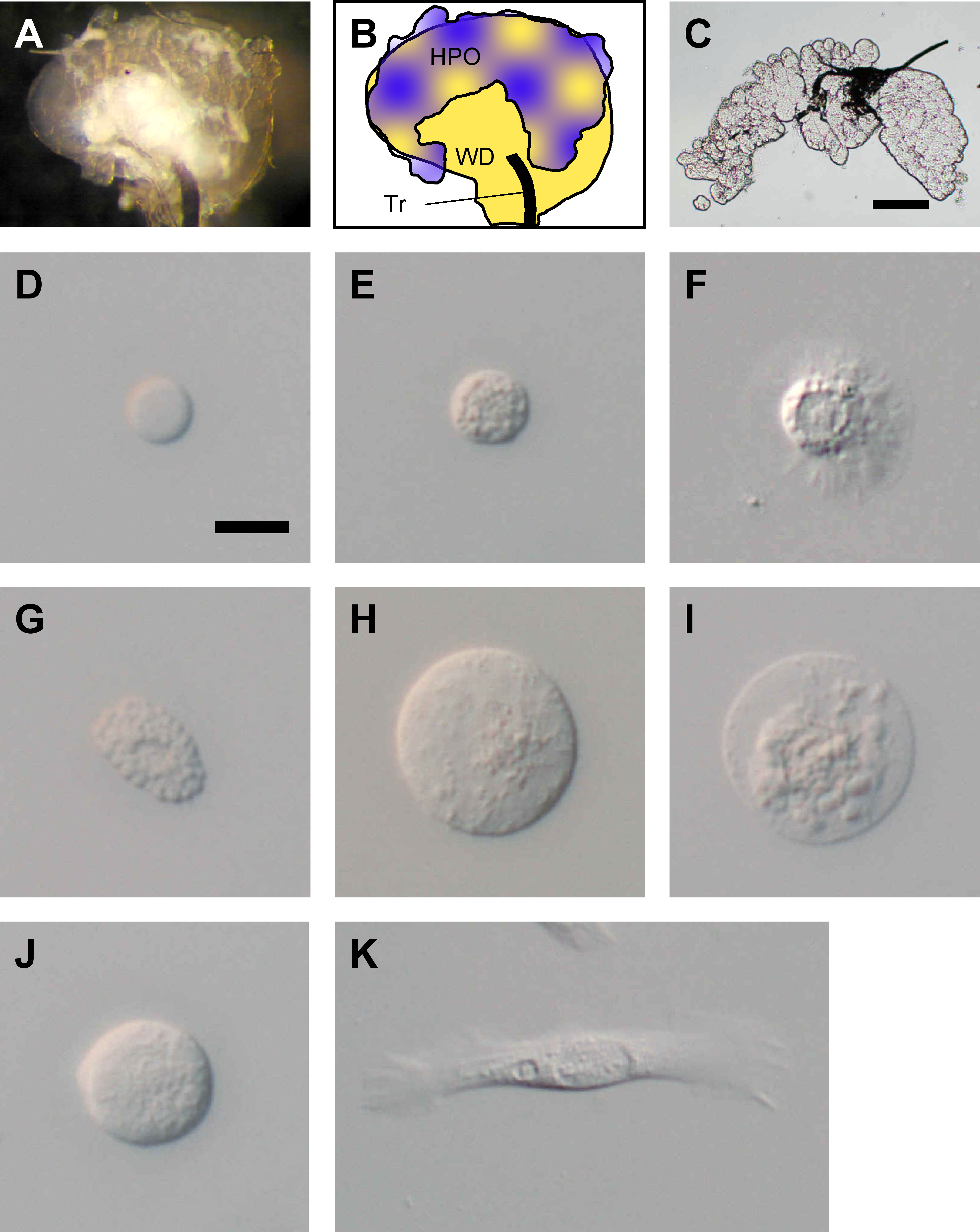

Supplement: Figure S1 — Hematopoietic organ (A–C) and circulating hemocytes (D–K) in B. mori (standard line). A: Hematopoietic organ (HPO) attached to imaginal fore wing disc from L5D1 larva. B: an illustration of HPO (corresponding to panel A). C: an HPO separated from imaginal wing disc. Bars are 0.2 mm. Circulating hemocytes were classified into 5 morphotypes: prohemocytes (D), granulocytes (E), spherulocytes (G), oenocytoids (H), and plasmatocytes (J), by morphological criteria according to Akai and Sato [28], [30]. One hour after separation, granulocytes and plasmatocytes transformed into the spread form (F and K, respectively). Soon after isolation, oenocytoids collapsed to release pro-PO (I). (5.88 MB TIF) [file pone.0011816.s001.tif]

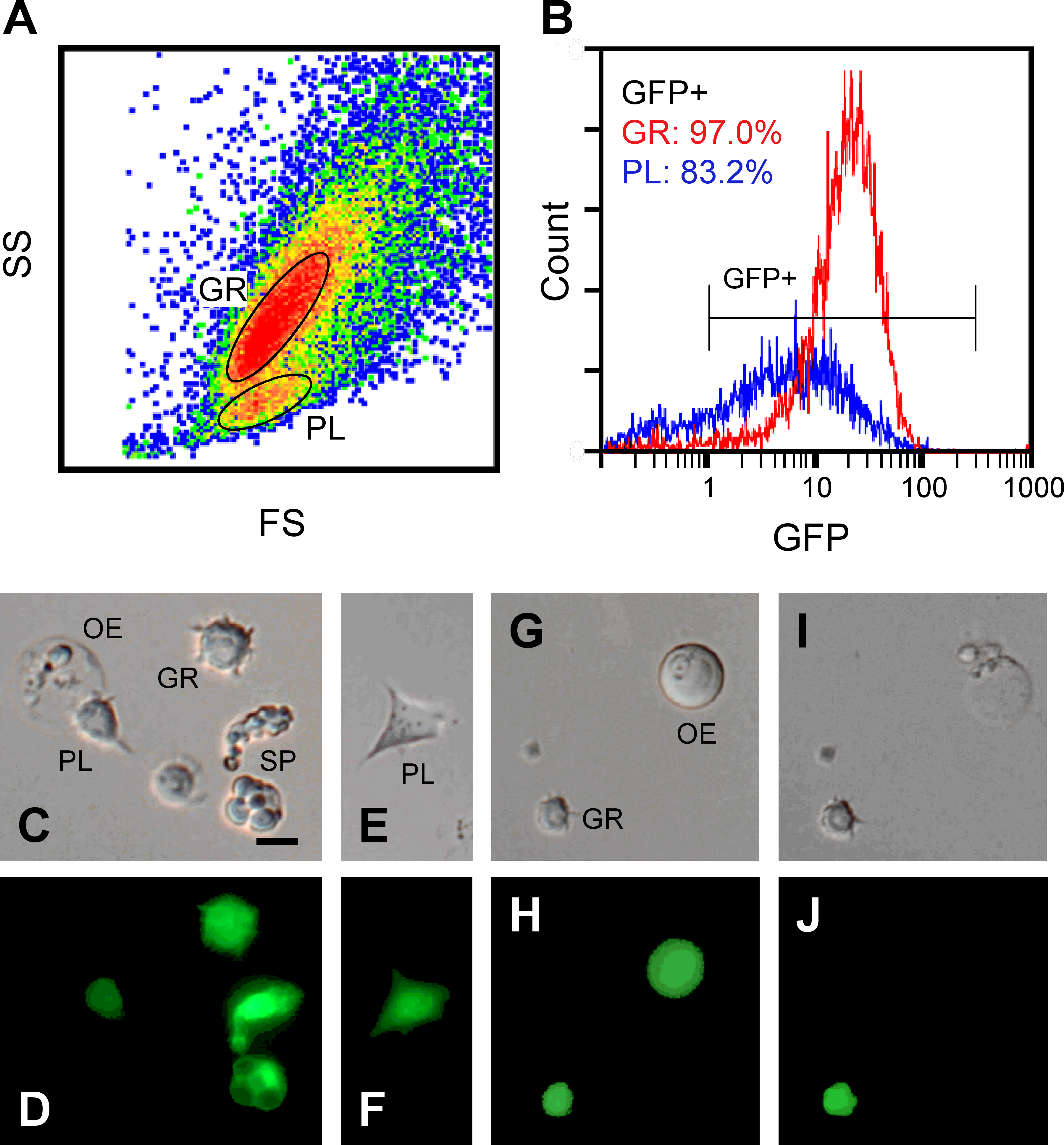

Supplement: Figure S2 — Hemocytes from CecB-GFP larvae. A,B: flow cytometric analysis. A: granulocytes and plasmatocytes are dominant in silkworm, and were roughly divided on two-dimensional plots with FS/SS [9]. B: 97% of granulocytes (GR) and 83% of plasmatocytes (PL) express GFP. C–F: CecB-GFP hemocytes viewed under a differential interference microscope (upper) and a fluorescent microscope (lower) immediately after isolation (C–H) and a few minutes later (I, J). PL and GR fluoresce bright green (D, F). An oenocytoid (OE) initially looks bright (G, H) but soon after isolation collapsed and turned dark (J: the same frame as panel H, 3 min later). Spherulocytes (SP) fluoresce green in the nucleus and cytoplasm but not in the spherules. All photographs are at the same magnification (bar = 10 µm). (2.49 MB TIF) [file pone.0011816.s002.tif]

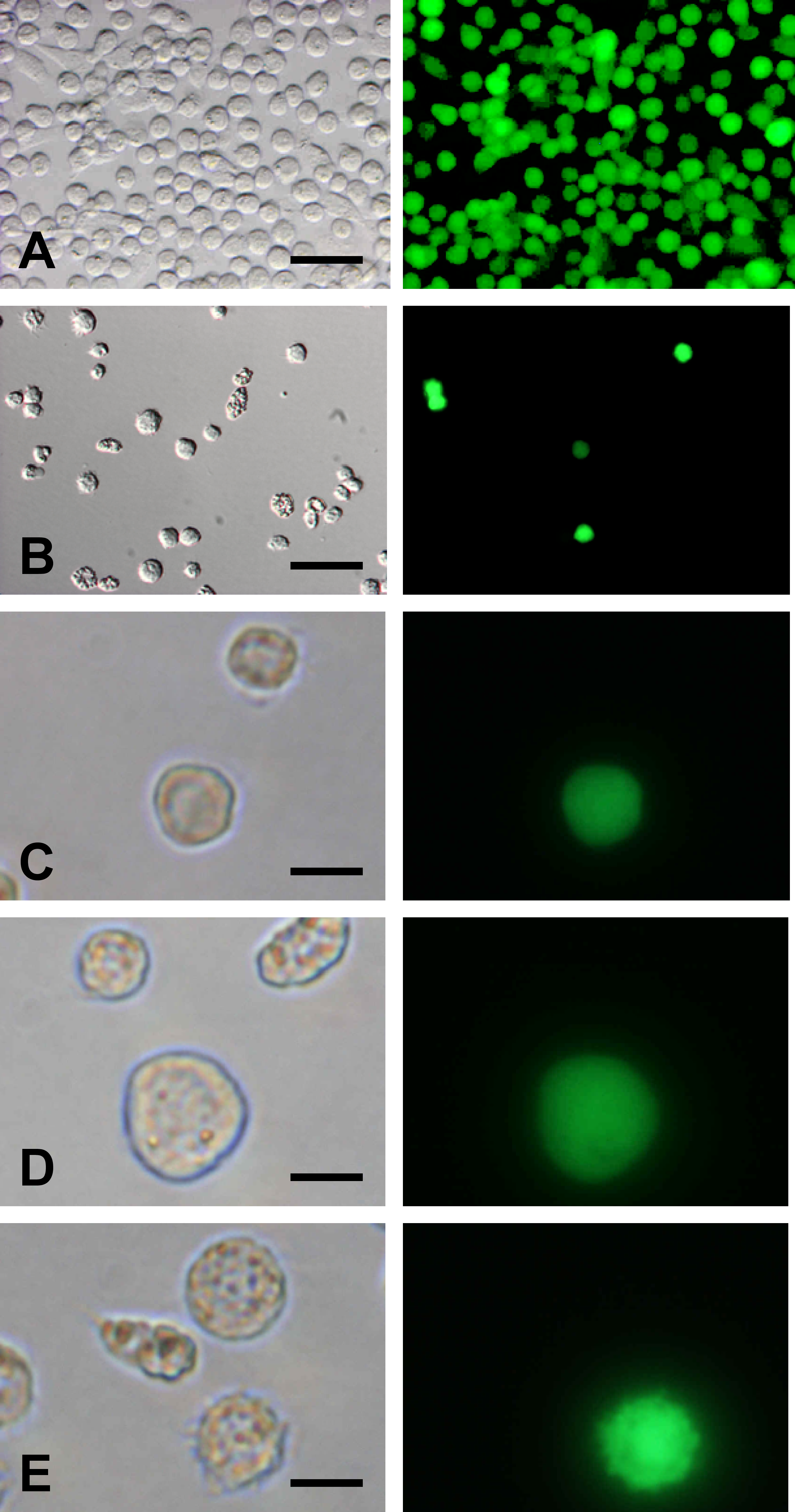

Supplement: Figure S3 — Hemocytes derived from HPO cells after in vivo differentiation. HPOs from CecB-GFP larvae were enzymatically dispersed (A) and injected into non-transgenic larvae. Five days later, cells were recovered and viewed under a differential interference microscope (left) and a fluorescent microscope (right). Cells derived from implanted HPO cells expressed GFP (B). C: GFP-expressing plasmatocyte. D: GFP-expressing oenocytoid. E: GFP-expressing granulocyte. Bar = 40 µm (A, B), 10 µm (C, D, E). (3.47 MB TIF) [file pone.0011816.s003.tif]

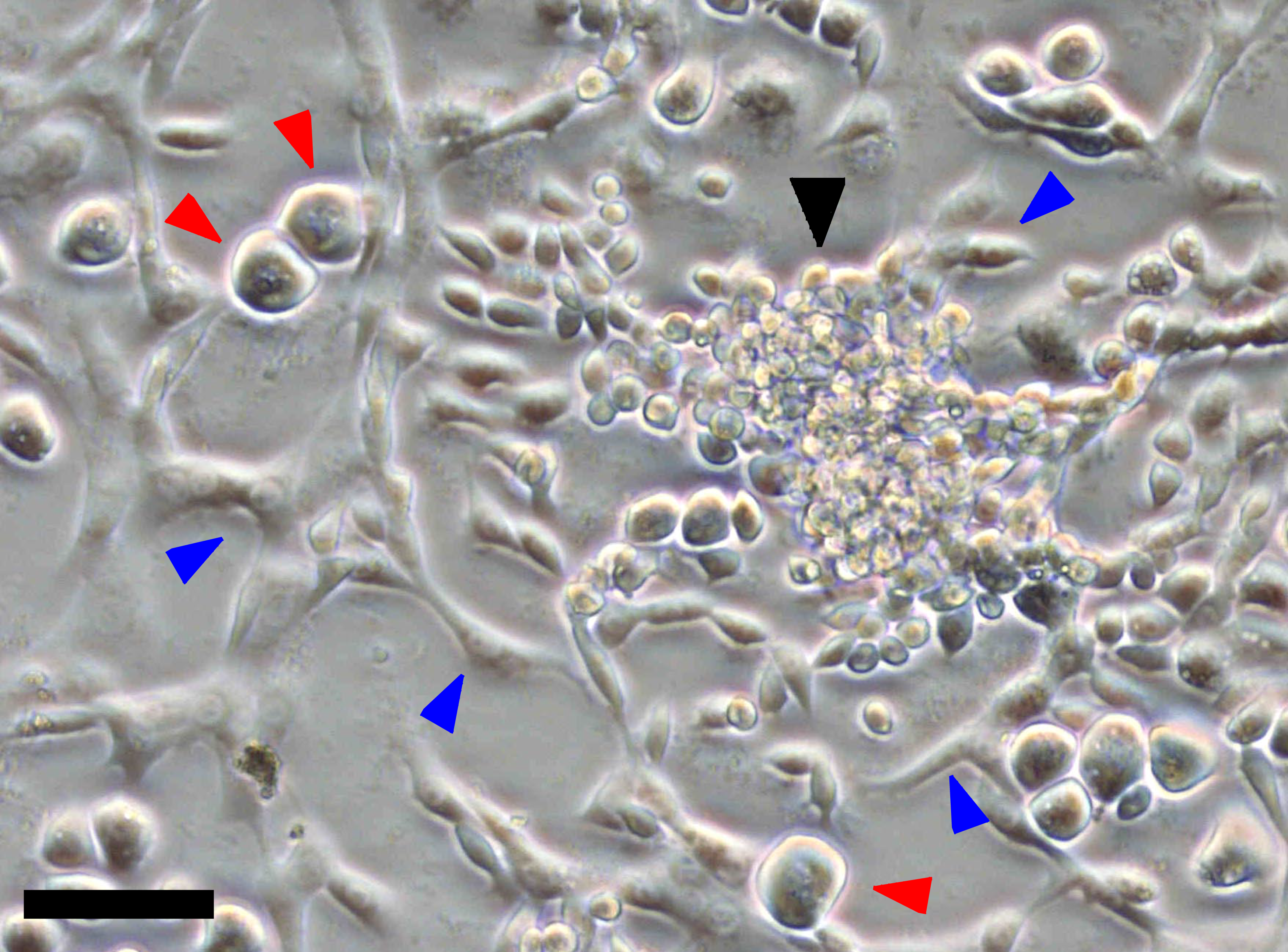

Supplement: Figure S4 — Culture of HPO cells. Enzymatically-dispersed HPO cells were cultured with 3% larval plasma for 4 days. Black arrowheads: a mass of small prohemocyte-like cells. Blue arrowheads: spread plasmatocyte-like cells. Red arrowheads: large oenocytoid-like cells. Bar = 50 µm. (8.38 MB TIF) [file pone.0011816.s004.tif]
